# Lipid variability and risk of microvascular complications in patients with diabetes: a systematic review and meta-analysis

**DOI:** 10.1186/s12902-023-01526-9

**Published:** 2024-01-02

**Authors:** Mohammad Amin Karimi, Ali Vaezi, Akram Ansari, Iman Archin, Kiarash Dadgar, Asma Rasouli, Parna Ghannadikhosh, Goharsharieh Alishiri, Neda Tizro, Fatemeh Gharei, Saba Imanparvar, Sakineh Salehi, Seyed Amirhossein Mazhari, Mohammad Hossein Etemadi, Milad Alipour, Niloofar Deravi, Mahdyieh Naziri

**Affiliations:** 1 School of Medicine, Shahid Beheshti University of Medical Sciences, Tehran, Iran; 2 Student Research Committee, School of Medicine, Tehran University of Medical Sciences, Tehran, Iran; 3 Medical Student, Shantou University Medical College, Shantou, Guangdong China; 4 Kazan (Volga Region) Federal University, Kazan, Russia; 5 Young Researchers Elite Club, Islamic Azad University Tehran Medical Branch, Tehran, Iran; 6 School of Medicine, Zanjan University of Medical Sciences, Zanjan, Iran; 7 Student Research Committee, Tabriz University of Medical Sciences, Tabriz, Iran; 8 Students Research Committee, School of Medicine, Ardabil University of Medical Sciences, Ardabil, Iran; 9 Student Research Committee, School of Medicine, Guilan University of Medical Sciences, Rasht, Iran; 10 School of Medicine, Ardabil University of Medical Sciences, Ardabil, Iran; 11 Department of Medicine, Ardabil Medical Sciences Branch, Islamic Azad University, Ardabil, Iran; 12 Student Research Committee, Azerbaijan Medical University, Baku, Azerbaijan; 13 School of Medicine, Isfahan University of Medical Sciences, Isfahan, Iran; 14 Medical Student, Department of Medicine, Islamic Azad University Tehran Medical Sciences, Tehran, Iran; 15 Students Research Committee, School of Medicine, Iran University of Medical Sciences, Tehran, Iran

**Keywords:** Retinopathy, Neuropathy, Nephropathy, Lipid variability, Microvascular complication

## Abstract

**Background and aims:**

The current systematic review aimed to elucidate the effects of lipid variability on microvascular complication risk in diabetic patients. The lipid components studied were as follows: High-density lipoprotein (HDL), High-density lipoprotein (LDL), Triglyceride (TG), Total Cholesterol (TC), and Remnant Cholesterol (RC).

**Method:**

We carried out a systematic search in multiple databases, including PubMed, Web of Science, and SCOPUS, up to October 2nd, 2023. After omitting the duplicates, we screened the title and abstract of the studies. Next, we retrieved and reviewed the full text of the remaining articles and included the ones that met our inclusion criteria in the study.

**Result:**

In this research, we examined seven studies, comprising six cohort studies and one cross-sectional study. This research was conducted in Hong Kong, China, Japan, Taiwan, Finland, and Italy. The publication years of these articles ranged from 2012 to 2022, and the duration of each study ranged from 5 to 14.3 years. The study group consisted of patients with type 2 diabetes aged between 45 and 84 years, with a diabetes history of 7 to 12 years. These studies have demonstrated that higher levels of LDL, HDL, and TG variability can have adverse effects on microvascular complications, especially nephropathy and neuropathic complications. TG and LDL variability were associated with the development of albuminuria and GFR decline.

Additionally, reducing HDL levels showed a protective effect against microalbuminuria. However, other studies did not reveal an apparent relationship between lipid variations and microvascular complications, such as retinopathy. Current research lacks geographic and demographic diversity. Increased HDL, TG, and RC variability have been associated with several microvascular difficulties. Still, the pathogenic mechanism is not entirely known, and understanding how lipid variability affects microvascular disorders may lead to novel treatments. Furthermore, the current body of this research is restricted in its coverage. This field's lack of thorough investigations required a more extensive study and comprehensive effort.

**Conclusion:**

The relationship between lipid variation (LDL, HDL, and TG) (adverse effects) on microvascular complications, especially nephropathy and neuropathic (and maybe not retinopathy), is proven. Physicians and health policymakers should be highly vigilant to lipid variation in a general population.

## Introduction

Over 450 million people are dealing with diabetes worldwide, and this number is increasing year by year. According to the International Diabetes Federation Atlas, by 2045, there will be 700 million patients with diabetes worldwide [1]. There are a variety of diabetes complications, and microvascular complications are one of the most important ones.

The most critical microvascular complications of diabetes are nephropathy, neuropathy, and retinopathy, which are responsible for a significant increase in morbidity and mortality of patients with diabetes [2, 3]. Around 20–40% of patients with diabetes experience diabetic nephropathy; therefore, due to their population, the most common cause of chronic kidney disease (CKD) is diabetes mellitus [4, 5]. Diabetic neuropathy happens in half of the patients with diabetes in a lifetime and is the leading cause of lower extremity amputation [6, 7]. Diabetic retinopathy develops in 10% of patients with diabetes, and in developed countries, it is the most common cause of blindness in the 15–64 years old population [8, 9].

It is common among type 2 individuals with diabetes to be dyslipidemic, even with reasonable control of glycemic indices [10]. Higher lipid variability has been linked to poorer health outcomes in both diabetic and non-diabetic populations [11–13]. Lipid variability has been studied in several articles as the variation of high-density lipoprotein (HDL), low-density lipoprotein (LDL), triglyceride (TG), cholesterol, and apolipoprotein between visits. Many studies have analyzed lipid variability in patients with diabetes and their effect on cardiovascular diseases [14, 15] or their mortality [16, 17], and some studies showed that an abnormal lipid profile could cause an increase in the risk of developing diabetes complications such as microvascular complications-mostly neuropathy and nephropathy [18–20]. In a cohort study by Wang et al., mortality risk was significantly increased with greater LDL, HDL, and total cholesterol (TC) variability in patients with type 2 diabetes. HDL variability was related to non-cardiovascular deaths [21]. Another study found no association between diabetic retinopathy and lipid variability. Still, the study showed that higher TG, HDL, and cholesterol levels increase the risk of developing nephropathy and neuropathy [22]. Brandini et al. found that TG variability is associated with microalbuminuria incidence in a sample of 457 patients with type 2 diabetes [11]. Also, another study of 846 individuals with type 2 diabetes showed HDL variability is related to diabetic nephropathy risk increase [23].

Few studies have focused on the association between lipid fluctuations and diabetic microvascular complications. In the past decade, the vast majority of studies investigating the association between lipid variability and microvascular complications have been limited to examining specific lipid parameters or particular microvascular complications [24, 25]. Controversies exist among the results of studies on the effect of lipid variability on complications of diabetes. To the best of our knowledge, this study aims to conduct a first-of-its-kind evaluation by systematically examining the relationship between lipid variability and susceptibility to microvascular complications among individuals with diabetes.

## Methods

### Protocol and registration

We conducted the current systematic review according to the Preferred Reporting Items for Systematic Reviews and Meta-Analyses (PRISMA) statement [26]. The protocol for the study has been filled out in the Open Science Framework (https://osf.io/yjns6).

### Search strategy

This systematic review was conducted in databases of PubMed, Web of Science, and SCOPUS (up to October 2nd, 2023) to detect the relevant studies.

A combination of keywords and Medical Subject Headings (Mesh) phrases (retinopathy, neuropathy, nephropathy, kidney, CKD, chronic kidney disease, micro, microvascular, apolipoprotein, lipoprotein, HDL, high-density lipoprotein, LDL, low-density lipoprotein, Triglycerides, Cholesterol, variation, and variability) were used in the search strategy. The complete search strategy applied to all databases is described in Table 1. No consideration was given to language restrictions.

**Table 1:** The Search Strategy in PubMed, Scopus, and Web of Science Databases Conducted on October 2nd, 2023

Search Engine	Search Strategy	Results
SCOPUS	TITLE-ABS-KEY ( ( retinopathy OR "Retinal Disease*" OR neuropathy OR nephropathy OR "kidney disease*" OR ckd OR "microvascular complication*") AND ( apolipoprotein* OR lipoprotein* OR hdl OR ldl OR triglyceride* OR triacylglycerol* OR epicholesterol* OR cholesterol*) AND ( variation* OR variability*))	1710
PubMed	(retinopathy[Title/Abstract] OR "Retinal Diseases"[Mesh] OR "Retinal Disease"[tiab] OR neuropathy[Title/Abstract] OR "Kidney Diseases"[Mesh] OR nephropathy[Title/Abstract] OR kidney[Title/Abstract] OR CKD[Title/Abstract] OR kidney disease[Title/Abstract] OR "microvascular complication"[Title/Abstract]) AND ("Apolipoproteins"[Mesh] OR apolipoprotein[Title/Abstract] OR "Lipoproteins"[Mesh] OR lipoprotein[Title/Abstract] OR "Lipoproteins, HDL"[Mesh] OR HDL[Title/Abstract] OR "Lipoproteins, LDL"[Mesh] OR LDL[Title/Abstract] OR "Triglycerides"[Mesh] OR Triglyceride*[Title/Abstract] OR Triacylglycerol*[tiab] OR "Cholesterol"[Mesh] OR Epicholesterol[tiab] OR Cholesterol*[Title/Abstract]) AND (variation[Title/Abstract] OR variability[Title/Abstract])	547
Web of Science	(TI = ( retinopathy OR "Retinal Disease*" OR neuropathy OR "Kidney Disease*" OR nephropathy OR CKD OR "microvascular complication*") OR AB = ( retinopathy OR "Retinal Disease*" OR neuropathy OR "Kidney Disease*" OR nephropathy OR CKD OR "microvascular complication*")) AND (TI = ( Apolipoprotein* OR lipoprotein* OR HDL OR LDL OR Triglyceride* OR Triacylglycerol* OR Cholesterol* OR Epicholesterol*) OR AB = ( Apolipoprotein* OR lipoprotein* OR HDL OR LDL OR Triglyceride* OR Triacylglycerol* OR Cholesterol* OR Epicholesterol*)) AND (TI = (Variation OR variabilit*) OR AB = ( Variation OR variabilit*))	297

### Eligibility criteria

We included observational studies (cross-sectional, cohort, Case reports, and Case series) that met the following inclusion criteria: Observational studies on the effect of lipid variability on microvascular complications in patients with diabetes.

The exclusion criteria were as follows: review articles, editorials, commentaries, in vivo, in vitro, and randomized clinical trial studies. Additionally, more relevant studies were found by manually looking through the references of publications in the initial search.

### Study selection

Following the removal of duplicate records, each title and abstract were independently reviewed by two reviewers (Mohammad Amin Karimi and Kiarash Dadgar). Disagreements were settled by consulting a third reviewer or reaching a consensus (Fatemeh Gharei). Studies that matched the criteria for inclusion had their complete texts retrieved and were subjected to an independent analysis by two writers (Mohammad Amin Karimi and Neda Tizro). A third author (Niloofar Deravi) was consulted when reviewers could not agree. Studies that did not fit the inclusion criteria were ultimately eliminated.

**Fig. 1:**
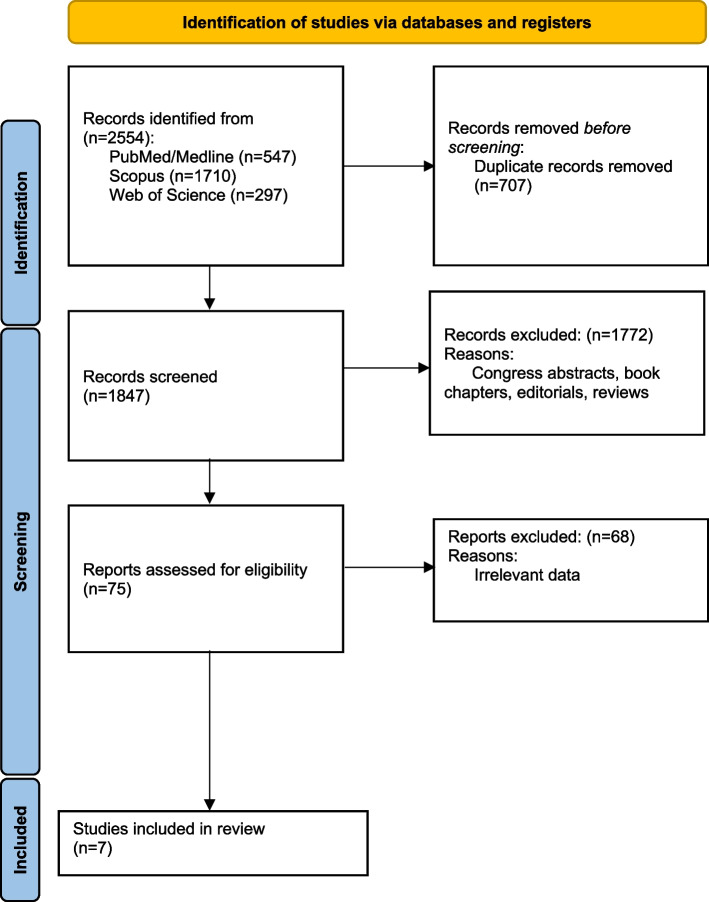
The PRISMA chart of the current systematic review

The 2020 PRISMA checklist, depicted in Fig. 1, demonstrates the screening procedure.

### Quality assessment

Two reviewers independently assessed the quality and bias risk of all the studies that satisfied the inclusion criteria using the Joanna Briggs Institute (JBI) Critical Appraisal tools (https://jbi.global/critical-appraisal-tools) [27].

This instrument assessed the reporting or methodology of all types of studies. Ten questions comprise the JBI tool for qualitative studies; each has four possible answers: yes, no, unclear, and not applicable. Each "yes" response results in a score, and if 70% of the questions in a study were answered "yes," bias risk was assumed to be "low"; if 50% to 69% of the questions were answered "yes," bias risk was evaluated to be "moderate", and if less than 50% of the questions were answered "yes," bias risk was assumed to be "high". Conflicts were settled through consensus.

### Data extraction

Using an established standardized template, two reviewers (Mohammad Amin Karimi and Kiarash Dadgar) separately retrieved the following data from the included articles: Author and publication year, country, study design, follow-up duration, population and gender, definition of lipid variabilities and adjustments, and outcomes. A third author (Niloofar Deravi) was consulted in cases of disagreement between reviewers.

## Results

### Literature search

For this systematic review, 2554 studies were identified through a primary literature search in Scopus, PubMed, and Web of Science databases. After omitting the duplicates, a total of 1847 studies were left. Among them, 1772 cases did not apply to the purpose of the study and, therefore, were excluded by title/abstract screening. Subsequently, 75 potentially relevant records were subjected to full-text review. Of these, 68 cases were also removed because of irrelevant data. The database search method is summarized in Table 1.

### Study characteristics

Finally, seven articles with a total population of 144,226 were reviewed. Six of these seven observational studies were cohort research [11, 23, 28–31], and one was cross-sectional [22]. This research was conducted in China [22], Japan [29], Hong Kong [30], Taiwan [23], Finland [31], and Italy [11, 28]. The average age of the patients varied from 45 to 84 years. The follow-up duration of cohort studies ranged from 5 to 14.3 years.

Regarding the quality assessment, using the JBI critical appraisal forms [27], the score among the included cohorts ranged from 9/11 to 11/11, and the single cross-sectional study scored 8/8, rendering the total bias of the included studies as “low”.

In these seven studies, the effects of lipid variability on microvascular complication risk in patients with diabetes were assessed. The lipid components studied were as follows: LDL [22, 23, 28, 30], HDL [22, 23, 28, 30], TG [11, 22, 23, 28–30], TC [28], RC [22, 31].

These studies have shown that higher LDL, HDL, and TG variability adversely affect microvascular complications, especially nephropathy and neuropathic complications [30]. TG and LDL variability was associated with developing albuminuria and estimated glomerular filtration rate (eGFR) decline [28, 29]. In another study, lower levels of HDL variation had a protective effect on microalbuminuria [23]. In contrast, another study has shown no evidence of a relationship between lipid variation and microvascular complications such as retinopathy [22]. Table 2 displays a comprehensive summary of the data extracted from the studies that have been incorporated.

**Table 2:** Detailed summary of the extracted data from the included studies

Author, Year	Country		Type of Study	Follow-up duration (years)	Population	Sex (female)	Lipid variability definition	Adjustments	Outcomes	Quality score
Ceriello et al. 2017 [28]	Italy	Retrospective Cohort		7	Investigation of albuminuria and reduction of eGFR (< 60 ml/min/1.73m^2^) in 4231 and 7560 participants, respectively	49.1% of females with albuminuria 40.6% of females with developed eGFR	The median and IQR of LDL and HDL	Age, gender, duration of diabetes, smoking status, BMI, hypertension, and values of HbA1c, SBP, DBP, serum UA, total cholesterol, HDL, LDL, TG, and eGFR, and medication intake (metformin, thiazolidinedione, sulfonylurea, glinides, GLP-1 analogs, DPP-IV inhibitors, insulin, statins, aspirin, ACEI, and ARBs)	1. Positive association of albuminuria risk with HbA1c variability (upper quartile HR = 1.3; 95%CI = [1.1–1.6]) 2. The greatest risk of albuminuria is in the simultaneous variation of HbA1c and HDL (HR = 1.47; 95%CI = [1.17, 1.84]) 3. Positive correlation of variability in SBP, DBP, HDL, LDL, and especially UA (upper quartile HR = 1.8; 95%CI = [1.3–2.4]) with the reduction of eGFR 4. The greatest risk of eGFR reduction is in high variability of UA (HR = 1.54; 95%CI = [1.19, 1.99]) and DBP (HR = 1.47; 95%CI = [1.11–1.94])	9/11
Chang et al. 2012 [23]	Taiwan	Retrospective Cohort		5	2711 participants with T2D	56%	SD of HDL and LDL and TG	Age, gender, smoking status, disease duration, baseline albuminuria stage, baseline serum creatinine level, ACEI, ARB, statin and fibrate, DBP, HDL, LDL, TG	1. Observation of higher mean HDL as a protective factor against DN progression (HR = 0.97, 95%CI = [0.95, 0.98], *P* = 0.002) 2. The positive relationship between HDL variations and the risk of developing DN (HR = 1.17, 95%ci = [1.03, 1.341], *P* = 0.015] 3. The lowest risk of DN at a higher level and less variability of HDL	11/11
Matsuoka-Uchiyama et al. 2022 [29]	Japan	Retrospective Cohort		7	527 participants with Type2 DM	42%	SD, Adj-SD, and MMD of TG, LDL, HDL	Age, sex, BMI, mean TG, baseline eGFR, proteinuria, HbA1c, smoking, hypertension, fibrates intake	1. There is a significant positive relationship between lower values of SD, Adj-SD, and MMD with increased renal survival in the adjusted model (HR, 1.62, 1.66, and 1.59; 95%CI = [1.05, 2.53], [1.08, 2.58], [1.04, 2.47], respectively) 2. There is a significant relationship between lower values of SD, Adj-SD, and MMD with the absence of albuminuria	10/11
Hukportie et al. 2022 [22]	China	Cross-sectional		-	18,038 participants with DM (10,632 no case)	38%	SD, CV, and VIM of total cholesterol, LDL, HDL	Age, sex, race, allocation to glycemia treatment, arm blood pressure vs. lipid treatment, duration of diabetes, mean HbA1c, mean LDL, mean HDL, mean TG, mean SBP, baseline eGFR, baseline BMI, cardiovascular disease history, antihypertensive use, insulin, statin, fibrate, and other lipid medication	1. Higher levels of HDL, TG, and RC diversity were associated with a 57%, 50%, and 40% increased risk of diabetic nephropathy and a 36%, 47, and 15% increased risk of diabetic neuropathy, respectively 2. Lack of association between LDL and other lipids variability with microvascular complications	8/8
Wan et al. 2021 [30]	Hong Kong	Retrospective cohort		5	105 552 patients aged 45–84 with type 2 diabetes mellitus and normal kidney function	52.7%	SD of LDL, SD of TC to HDL ratio, SD of TG	Age, gender, duration of Diabetes Mellitus, smoking status, BMI, SBP, DBP, HbA1c, eGFR, urine albumin to creatinine ratio, the usages of anti-diabetic drugs, antihypertensive drugs, statins and fibrates, The Charlson index and usual LDL, TC to HDL ratio or TG	1. Each unit increase in LDL variability was associated with a 20%, 38%, and 108% higher risk of kidney disease, reduced renal function, and ESRD, respectively 2. Each unit increase in total cholesterol to HDL ratio variability was associated with a 35%, 33%, and 75% higher risk of kidney disease, reduced renal function, and ESRD, respectively	11/11
Jansson Sigfrids et al. 2021 [31]	Finland	Prospective cohort		8 (for DN) and 14.3 (for SDR)	5150 patients with type 1 diabetes	-	CV of Remnant cholesterol	diabetes duration, sex, HbA1c, systolic blood pressure, smoking status, body mass index, and estimated glucose disposal rate	1. Remnant cholesterol variability was not independently associated with DN progression and development of SDR	9/11
Bardini et al. 2016 [11]	Italy	Retrospective cohort		6.8	457 normoalbuminuric outpatients with type 2 diabetes	-	SD of TG, adj-SD of TG, Log of TG-SD, Adj-Log of TG-SD	HbA1c-mean, HbA1c-SD, and LogTG-mean	1. Higher median TG-SD (33.6 vs 29.0 mg/dl) and adj-TG-SD (31.4 vs 26.7 mg/dl) were significantly associated with increased incidence of microalbuminuria 2. LogTG-SD and adj-LogTG-SD were significant predictors of microalbuminuria (HR = 2.1, 1.5 and 95%CI = [1.1, 4.2], [1.1, 3.3], respectively)	11/11

***Abbreviations*** **:** *ACEI* Angiotensin-converting enzyme inhibitor, *AMD* Association of Medical Diabetologists, *ARB* Angiotensin receptor blocker, *BMI* Body mass index, *CI* Confidence interval, *CV* Coefficient of variation, *DBP* Diastolic blood pressure, *DM* Diabetes mellitus, *DN* Diabetic nephropathy, *DPP-IV* Dipeptidyl peptidase-IV, *eGFR* Estimated glomerular filtration rate, *GLP-1* Glucagon-like peptide 1, *HDL* High-density lipoprotein, *HR* Hazard ratio, *IQR* Interquartile range, *LDL* Low-density lipoprotein, *MMD* Maximum minus minimum difference, *RC* Remnant cholesterol, *SBP* Systolic Blood pressure, *SD* Standard deviation, *SDR* Severe diabetic retinopathy, *T2D* Type 2 diabetes, *TC* Total cholesterol, *TG* Triglyceride, *UA* Uric acid, *VIM* Variability independent of the mean

### Meta-analysis and publication bias

**Fig. 2:**
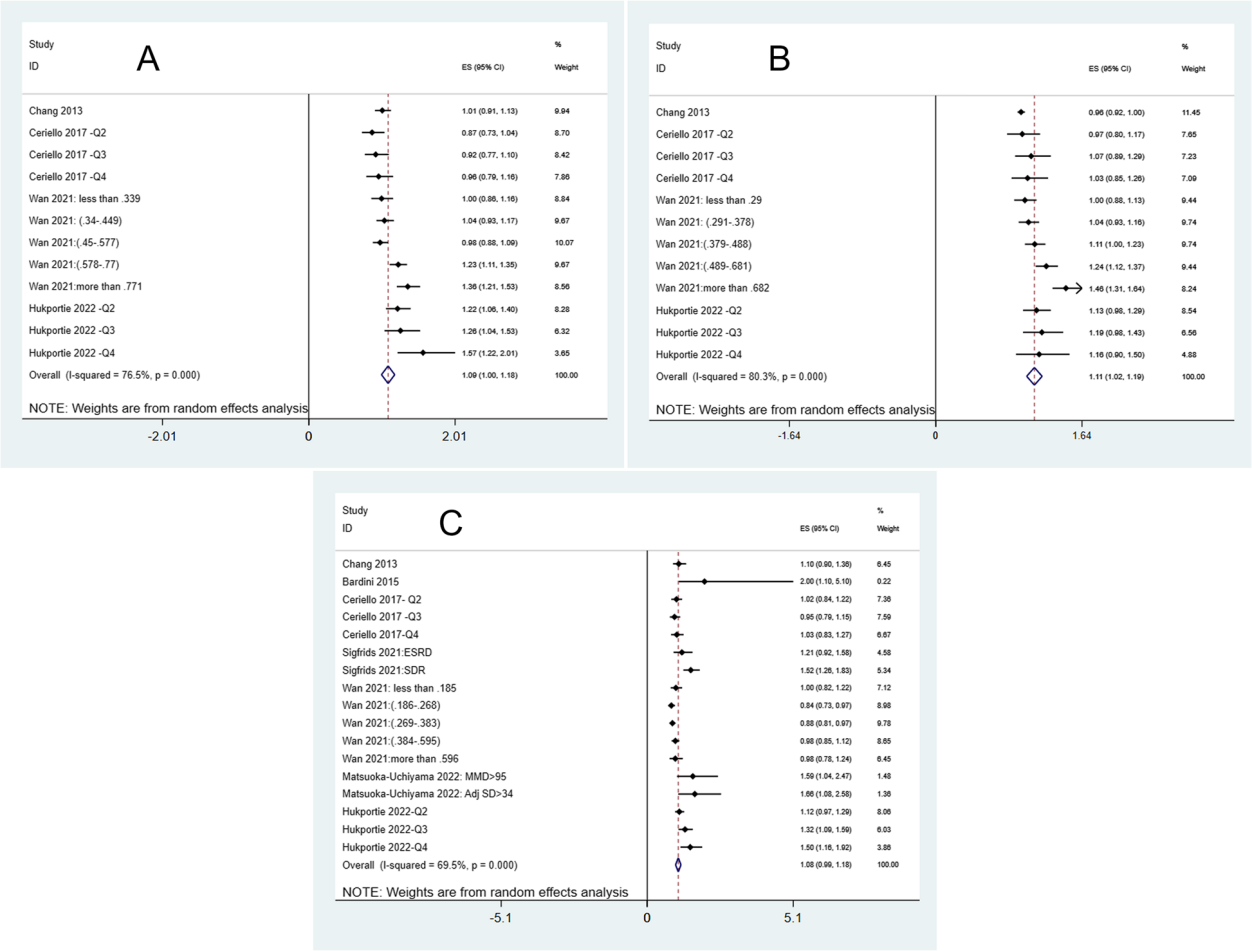
The forest plot for A) HDL B) LDL C) TG; HDL, LDL, and TG were significantly correlated with 9%, 11%, and 8% increases in risk of microvascular complications in diabetic patients, respectively. All these analyses showed moderate to severe heterogeneity

We performed a meta-analysis of 7 studies to quantify the association between lipids variability, including TG, LDL, and HDL variability, and microvascular complications risk in diabetic subjects. Figure 2 summarizes the results of this meta-analysis. A significant positive relationship was observed between the higher variability of different lipids and the risk of microvascular complications. So the increase of each unit in TG (OR = 1.08, 95%CI = [0.99, 1.18]), LDL (OR = 1.11, 95%CI = [1.02, 1.19]), and HDL (OR = 1.09, 95%CI = [1.00, 1.18]) was associated with an increase of 8%, 11% and 9% of microvascular complications, respectively. All of these associations were significant (*P* < 0 001). Moreover, medium to high heterogeneity of studies was reported, and its values for TG, LDL, and HDL were 69.5%, 80.3%, and 76.5%, respectively.

We evaluated the publication bias by Egger’s test, Begg’s test, and funnel plot. The funnel plot related to TG, LDL, and HDL was symmetrical, and these results were confirmed by Begg’s and Egger’s tests. Based on these results, there is probably no publication bias for the included studies.

## Discussion

Diabetes complications have become a great matter of health since the number of people with diabetes has been increasing yearly. Among them, microvascular complications are deemed to be one of the most important ones, including nephropathy, neuropathy, and retinopathy, which contribute to both morbidity and mortality of patients with diabetes [2, 3].

**Fig. 3:**
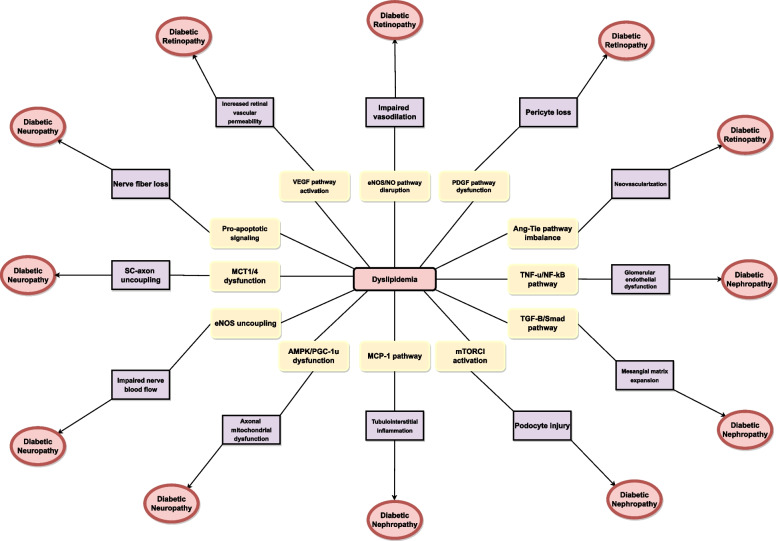
This diagram illustrates the impact of lipid alterations and heightened lipid concentrations, ultimately precipitating the development of diabetic microvascular complications. VEGF: Vascular endothelial growth factor, eNOS: Endothelial nitric oxide synthase, NO: Nitric oxide, PDGF: Platelet-derived growth factor, Ang-Tie: angiopoietin-Tie, TNF-α: Tumor Necrosis Factor-alpha, NF-kB: Nuclear factor kappa B, TGF-β: Transforming growth factor-β, mTORC1: mammalian target of rapamycin complex 1, MCP-1: Monocyte chemoattractant protein-1, AMPK: Adenosine monophosphate-activated protein kinase, PGC-1α: Peroxisome proliferator-activated receptor-gamma coactivator(PGC)-1alpha, SC-axon: Schwann cells axon, MCT: Monocarboxylate Transporter 1

Dyslipidemia in individuals with diabetes is a common phenomenon [10]. Studies have investigated the role of lipid variability in developing diabetic microvascular complications and showed relations between lipid profile variations and risk of nephropathy, neuropathy, and retinopathy [28, 30]. Figure 3 depicts how changes in lipid levels and increased lipid concentrations lead to the emergence of diabetic microvascular complications.

This systematic review of seven studies (144,226 diabetic patients) demonstrated the association between higher variability in lipid indices and a greater chance of developing microvascular complications (nephropathy, neuropathy, and retinopathy) in individuals with diabetes. Among the five included studies, different variability indices such as standard deviation (SD), adjusted standard deviation (Adj-SD), the maximum minus minimum difference (MMD), coefficient of variation (CV), and variability independent of the mean (VIM) were used.

### Nephropathy

Significant morbidity and mortality risks in people with type 1 and type 2 diabetes are attributable to diabetic kidney disease, which affects 30–40% of people with diabetes. Diabetic nephropathy is a significant contributor to End-Stage Renal Disease, and its initial sign is Microalbuminuria, which often results in macroalbuminuria, renal insufficiency, and hypertension [32]. The relationship between hyperlipidemia in individuals with diabetes mellitus and renal insufficiency has been established [33, 34], and renal damage and the nephrotic syndrome were both improved by lipid-lowering therapy in animal models [35, 36]. Ceriello et al. [28] found a higher HDL and LDL variability to be associated with poor renal outcomes, estimated glomerular filtration rate (eGFR) decline, and incidence of albuminuria; however, in their study, LDL variability was only associated with an increase in eGFR decline (not albuminuria), and TC variability showed no associations. It is essential to note the independence of low eGFR and albuminuria in terms of their risk factors. eGFR is more of a dynamic measurement of renal function, whereas albuminuria refers to fixed organ damage. Accordingly, it stands to reason that different conditions may not be equally affected by other lipid parameters [28].

Moreover, some studies illustrated the role of higher HDL variability in increased nephropathy risk. Hukportie et al. also used RC variability and showed a higher risk of poor renal outcomes with higher variability [22]. These findings were inconsistent with the result of another study [31], on individuals with type 1 diabetes and could be because of differences in lipid profiles of patients with type 1 and type 2 diabetes. Unexpectedly, Hukportie et al. [22]  and Chang et al. [37] found no association between LDL variability and nephropathy incidence. This finding could result from the patients’ aggressive LDL level control that diminished the adverse effects of high LDL variability [22]. Also, in the Wan et al. [30] study, an increase in LDL and TC to HDL ratio variability was associated with a higher risk of kidney disease, renal function decline, and end-stage renal disease. The reason for adopting the TC to HDL ratio in Wan et al. [30] study was better predictability than the other lipid variability measures, especially in an elderly study sample [38].

Hukportie et al. [22] reported an increased risk of nephropathy with higher TG variability. Bardini et al. [11] examined the relationship between TG variability and the incidence of microalbuminuria in 457 patients with type 2 diabetes, and they found that an increased intra-individual triglyceride variability has been identified as a prognostic factor for the development of microalbuminuria and nephropathy in individuals with type 2 diabetes. Similarly, Matsuoka-Uchiyama et al. [29] found postprandial TG to be a novel risk factor for microalbuminuria incidence and eGFR decline. However, Wan et al. [30], as well as Chang et al. [37], and Ceriello et al. [28] did not find any association between TG variability and nephropathy in individuals with type 2 diabetes [30]. The reason for this discrepancy is unclear, and no evidence has been found that fasting lipid profile assessments are superior to postprandial evaluations [29].

The mechanism by which high HDL, TG, and RC variability affect renal function has yet to be well known. Some studies have found hypertriglyceridemia to provoke inflammatory cytokines and free radicals generation, accentuating atherogenesis and endothelial damage, possibly contributing to albuminuria development [29]. However, suggestions point to the role of inflammation, oxidative stress (either by generating more free radicals or decreasing HDL protective actions), and vascular damage in altering the normal molecular signaling required for normal physiologic kidney function [22, 28].

As for LDL variability, it is suggested that higher variability in LDL levels could increase atherosclerosis by disrupting the normal endothelial function, inhibiting lipid efflux from the plaques, and disrupting the plaques. This mechanism has been explained for cardiovascular risks but could also reasonably explain the decline in renal function. One analysis [39] supported this hypothesis by demonstrating the relationship between TC to HDL ratio variability and the progression of atheroma volume. It has also been speculated that renal dysfunction could result from lipid variability acting as an epiphenomenon of conditions such as frailty. Lastly, low compliance to lipid-lowering medication such as statins has also been suggested to be a contributing cause of renal function decline [30].

### Neuropathy

Diabetic neuropathy is a prevalent contributor to both morbidity and mortality in individuals with diabetes. This particular kind of neuropathy is distinguished by symptoms such as pain, paresthesia, sensory impairment, a heightened susceptibility to falls, and a diminished quality of life for affected people [40, 41]. Dyslipidemia is a prevailing condition in individuals diagnosed with both type 1 diabetes mellitus and type 2 diabetes mellitus, and it exhibits an association with the development of diabetic neuropathy [42, 43]. Only one study investigated the role of lipid variability indices in developing diabetic neuropathy. Hukportie et al. showed that the risk of neuropathy increased with a higher quartile of HDL, TG, and RC variability, while the variability of LDL did not. Currently, there is not much data elucidating the relation between lipid variability parameters and the risk of peripheral neuropathy. In contrast, the effect of glycemic variability indices on neuropathy has been found and is well emphasized [22].

While a comprehensive understanding of the precise mechanisms underlying the impact of plasma lipids on diabetic nephropathy is still incomplete, it is plausible that many factors are implicated. To initiate, individuals grappling with dyslipidemia display insulin resistance and persistent inflammation, phenomena intricately linked with insulin resistance and potentially associated with peripheral neuropathy. Moreover, oxidative stress has emerged as a notable threat to DNA damage. Neuronal cells host receptors with the capacity to bind oxidized LDLs, initiating intricate cellular signaling pathways that culminate in the induction of oxidative stress. The role of oxidative stress, stemming from oxidized LDL, has been recognized in the genesis of nerve impairment within the context of dyslipidemia-associated neuropathy. Furthermore, the potential exists for lipid-induced nerve deterioration to trigger demyelination due to lipid profile alterations, a phenomenon inherently interlinked with diabetic neuropathy. Credible avenues interconnecting perturbations in lipid profiles with the progression of diabetic nephropathy encompass insulin resistance, inflammation, oxidative stress, and demyelination [44–51].

Also, the exact mechanism by which lipid variability influences neurons remains elusive; an intriguing hypothesis suggests that the normal functioning of mitochondria may be compromised due to disruptions in lipid metabolism resulting from dyslipidemia. These processes might lead to alterations in mitochondria size within the neurons of the dorsal root ganglion. Furthermore, demyelination has been proposed as an additional contributory mechanism to neuronal injury in lipid variations [48, 52].

### Retinopathy

Diabetic retinopathy is the primary cause of visual impairment in working-age people in developed nations[53]. The relationship between lipid levels and retinopathy is more complex. Many studies have shown the effect of lipoprotein (a) and TG on the progress and prognosis of diabetic retinopathy [54–56]. They have found a significant proportion of individuals with retinopathy have elevated Lipoprotein(a) levels compared to diabetic patients without retinopathy [56]. However, some other studies revealed that this relationship is insignificant [57]. Hukportie et al. found no associations between retinopathy incidence and any measure of lipid variability [22]. Sigfrids et al. found that the concentration of RC, but not its variability, is a predictive factor of diabetic retinopathy progression and development [31]. The pathophysiological mechanism and reason for this finding are unclear. It is suggested that the pathological processes may damage the eye slower than nephropathy and neuropathy, which require more time for detectable damage. As a result, it has been shown that the development of retinopathy is slower than the other two microvascular complications [22].

Our study had several strengths. This systematic review conducted a comprehensive search of multiple databases, thereby increasing the likelihood of identifying relevant studies and minimizing selection bias. In addition, our analysis considered various lipid components (LDL, HDL, TG, TC, and RC), providing a comprehensive view of how multiple aspects of lipid profiles may influence microvascular complications. This review examined a variety of microvascular complications, including nephropathy, neuropathy, and retinopathy, contributing to a deeper understanding of the relationship between lipid variability and diabetic complications.

However, our study has some limitations. Limited Geographic and Demographic Diversity is one of the shortcomings of current research. The studies included in this systematic review were conducted in specific regions, which may limit the applicability of the findings to a more diverse global population of patients with diabetes. The applicability of the results could be improved by incorporating studies from a wider variety of geographic regions and demographic groups. In addition, Increased HDL, TG, and RC variability have been linked to some microvascular complications; however, the underlying pathogenic process is unclear, and understanding the processes by which lipid variability impacts microvascular problems may lead to new treatment avenues. Future research could benefit from more standardized methodologies in order to enhance the comparability of results.

## Conclusion

Altogether, this systematic review highlighted the role of high lipid parameter variability in developing diabetic microvascular complications. There is still controversy about the predictive ability of some variability indices; therefore, more extensive studies could clarify such relationships. However, it is recommended that in lipid profile management of patients with diabetes, less variability be targeted, as it has shown a lower risk of developing microvascular complications.

## Data Availability

Data is available upon request from the corresponding author.

## Abbreviations

CKD: Chronic kidney disease
DNA: Deoxyribonucleic acid
eGFR: Estimated glomerular filtration rate
HDL: High-density lipoprotein
LDL: Low-density lipoprotein
RC: Remnant Cholesterol
TG: Triglyceride
